# Risk strata and quality of care for the elderly in Primary Health Care *

**DOI:** 10.1590/1518-8345.2968.3166

**Published:** 2019-10-07

**Authors:** Líliam Barbosa Silva, Patrícia Aparecida Barbosa Silva, Joseph Fabiano Guimarães Santos, Salete Maria de Fátima Silqueira, Eline Lima Borges, Sônia Maria Soares

**Affiliations:** 1 Universidade Federal de Minas Gerais, Escola de Enfermagem, Belo Horizonte, MG, Brasil.; 2 Bolsista da Coordenação de Aperfeiçoamento de Pessoal de Nível Superior (CAPES), Brasil.; 3 Hospital Governador Israel Pinheiro, Unidade de Terapia Intensiva, Belo Horizonte, MG, Brasil.

**Keywords:** Electronic Health Records, Quality of Health Care, Health of the Elderly, Primary Health Care, Chronic Disease, Cross-Sectional Studies, Registros Eletrônicos de Saúde, Qualidade da Assistência à Saúde, Saúde do Idoso, Atenção Primária à Saúde, Doença Crônica, Estudos Transversais, Registros Electrónicos de Salud, Calidad de la Atención de Salud, Salud del Anciano, Atención Primaria de Salud, Enfermedad Crónica, Estudios Transversales

## Abstract

**Objective:**

to identify patterns of associations between the degree of compliance to laboratory test requests by risk strata and the parameters of quality of care outcomes in primary health care (PHC).

**Method:**

a cross-sectional study involving 108 elderly patients with hypertension and/or diabetes treated in PHC. A semi-structured questionnaire and electronic medical record data were used. To evaluate the quality of care, the Patient Assessment of Chronic Illness Care (PACIC) questionnaire was used. Descriptive analysis, multiple correspondence analysis and k-means grouping were performed.

**Results:**

it was observed low compliance of the care practice, standing out as the worst parameter the evaluation of the diabetic foot (2.2%). Three clusters were identified, with cluster 1 having the highest number of individuals (37.0%), with better indicators of quality of care, evidenced by above 50% of compliance with laboratory tests (75.0%), high PACIC score (47.2%), control of blood pressure (70.0%) and metabolic levels (95.0%), and satisfaction with health (92.5%) and health access (90.0%). In contrast, cluster 3 (29.6%) was made up of individuals with worse outcomes of care.

**Conclusion:**

low compliance of care practice and asymmetries among health actions and users’ needs were observed, indicating failures in the care process in PHC.

## Abstract (pt)

**Objetivo:**

identificar padrões de associações entre o grau de conformidade às solicitações de exames laboratoriais por estratos de risco e os parâmetros de resultados da qualidade do cuidado na Atenção Primária à Saúde (APS).

**Método:**

estudo transversal envolvendo 108 idosos com hipertensão e/ou diabetes atendidos na APS. Utilizaram-se questionário semiestruturado e dados de prontuário eletrônico. Para avaliação da qualidade do cuidado, utilizou-se o questionário *Patient Assessment of Chronic Illness Care* (PACIC). Realizaram-se análise descritiva, análise de correspondência múltipla e agrupamento *k-means* .

**Resultados:**

observou-se baixa conformidade da prática assistencial, destacando-se como pior parâmetro a avaliação do pé diabético (2,2%). Foram identificados três *clusters* , sendo que o *cluster* 1 obteve maior número de indivíduos (37,0%), com melhores indicadores da qualidade do cuidado, evidenciado pelo cumprimento dos exames laboratoriais acima de 50% (75,0%), escore PACIC elevado (47,2%), controle dos níveis pressóricos (70,0%) e metabólico (95,0%), e satisfação com a saúde (92,5%) e com o acesso à saúde (90,0%). Em oposição, o *cluster* 3 (29,6%) foi constituído por indivíduos com piores indicadores de resultado do cuidado.

**Conclusão:**

constataram-se baixa conformidade da prática assistencial e assimetrias entre oferta de ações em saúde e necessidades dos usuários, indicando falhas no processo de cuidado na APS.

## Abstract (es)

**Objetivo:**

identificar patrones de asociación entre el grado de conformidad a las solicitudes de exámenes de laboratorio por estratos de riesgo y los parámetros de resultados de la calidad del cuidado en la Atención Primaria a la Salud (APS).

**Método:**

estudio transversal que involucra a 108 adultos mayores con hipertensión y/o diabetes atendidos en la APS. Se utilizaron cuestionarios semiestructurados y datos de prontuario electrónico. Para la evaluación de la calidad del cuidado, se utilizó el cuestionario *Patient Assessment of Chronic Illness Care* (PACIC). Se realizaron análisis descriptivos, análisis de correspondencia múltiple y agrupación *k-means* .

**Resultados:**

se observó baja conformidad de la práctica asistencial, destacándose la evaluación del pie diabético (2,2%) como peor parámetro. Se identificaron tres *clusters* , siendo que el cluster 1, obtuvo mayor número de individuos (37,0%), con mejores indicadores de la calidad del cuidado, evidenciado por el cumplimiento de los exámenes de laboratorio por encima del 50% (75,0%), puntuación PACIC elevada (47,2%), control de los niveles de presión (70,0%) y metabólico (95,0%), y satisfacción con la salud (92,5%) y con el acceso a la salud (90,0%). En oposición, el *cluster* 3 (29,6%) fue constituido por individuos con peores indicadores de resultado del cuidado.

**Conclusión:**

se constató baja conformidad de la práctica asistencial y asimetrías entre oferta de acciones en salud y necesidades de los usuarios, indicando fallas en el proceso de cuidado en la APS.

## Introduction

Diabetes *mellitus* (DM) and hypertension are currently among the most prevalent chronic conditions. Together, they are considered as the main primary causes of renocardiovascular diseases in the world scenario^1^ and responsible for 13.0 million deaths worldwide in 2015, of which 7.8 million were caused by complications of high blood pressure levels and 5.2 million due to metabolic uncontrol^2^ .

This scenario is alarming and suggests the need to invest in improvements in the quality of management of these morbidities, especially in Primary Health Care (PHC), since it is the level of attention responsible for the early detection of chronic conditions and monitoring of those conditions already diagnosed, avoiding complications and the need for hospitalization.

The Strategic Action Plan for Coping with Noncommunicable Chronic Diseases (NCD) in Brazil 2011-2022^3^ was adopted by the Ministry of Health as a guideline of actions directed at the care of the person who experiences a chronic health condition. The gateway to the Brazilian public health system ( *Sistema* Único *de Saúde* - SUS) consists of the Family Health Strategy, focused on primary health care and centered on the family and the community^4 - 7^ . Its main function is to provide person-centered care, with priority in actions of health promotion, disease prevention, health surveillance, assistance, and follow-up of the enrolled population, in an attempt to impact the quality of care provided to users with chronic conditions in PHC^3^ .

Some of the strategies recommended in this regard are related to the implementation of evidence-based clinical practices, as well as the monitoring of these interventions. However, although there is a high degree of agreement on the best practices of prevention, diagnosis and treatment of DM and hypertension, as described in several clinical guidelines and protocols, the use of these practices is still incipient, especially when it refers to the implementation of actions according to risk stratification of the population addressed. This becomes a barrier to achieve better care results based on current scientific knowledge^8 - 11^ .

A Brazilian study on the quality of care for the person with DM at a basic health unit in the city of Ribeirão Preto, São Paulo, based on 138 medical records, revealed that the actions implemented in the care are fragmented. No body mass index record was found; feet and ophthalmological assessment was recorded in only 15.2% and 4.3% of medical records, respectively; and only 1.4% of the results of laboratory tests had registered microalbuminuria levels^12^ .

These results would probably be more alarming if service provision according to the needs of the people addressed was taken into account. This was a concern already expressed by a scholar^13^ who alerted to the possibility that users with greater clinical risk might not be prioritized in the offer of actions to their needs, since health systems still chose planning of supply to the detriment of needs that is differentiated according to risk stratification. This aspect deserves attention, since, as a consequence, efforts and resources can be put in unnecessary, misguided, and inefficient interventions; reason why this issue has to be better explored.

Based on the above, this research hypothesizes that there are asymmetries between health care provision by the Family Health teams and the needs of the elderly with DM and/or hypertension, which implies a less favorable scenario for the segment with worse care results.

Currently there are not previous studies that have been conducted on the association between quality of care outcomes and providers’ compliance with care protocols for the elderly with DM and/or hypertension, considering the request for laboratory tests by risk stratification of individuals.

Therefore, the objective of this study was to identify patterns of associations between the degree of compliance to requests for laboratory tests by strata of cardiovascular risk and metabolic control, as well as the parameters of results of quality of care provided to the elderly with chronic conditions in PHC.

## Method

This cross-sectional study is part of the second phase of the population-based study on “Aging and Kidney Disease” (en-DoRen) at the regional level, conducted from August 2014 to January 2017 in one of the nine health districts of Belo Horizonte, Minas Gerais, Brazil^14^ .

The baseline sample from the “Aging and Kidney Disease” study is comprised of 300 randomized individuals. This analysis considered the data of the participants who met the following inclusion criteria: age ≥ 60 years; self-report DM and/or hypertension or having a confirmed diagnosis in an electronic medical record; having been followed for at least one year by the Family Health team of that district; having attended some type of health service of the basic health unit (BHU) in the last three years; having the electronic medical record located. The elderly with severe cognitive impairment (Mini-Mental State Examination - MMSE ≤ 9) were excluded from the analysis.

The sample size was based on conservative criteria, adopting a prevalence of 50% of the different outcomes (care parameters per cardiovascular risk stratum and metabolic control), which delimits the maximum variability of the sample size. Using the calculation method proposed by Lwanga and Lemeshow^15^ and considering absolute accuracy of 10%, with a significance level of 5%, a sample size of 96 individuals was found. Adopting 10% for possible losses, the total number was 106 people.

At the baseline of the “Aging and Kidney Disease” study, 143 PHC users were identified, of whom 118 were diagnosed with DM and/or hypertension and 10 participants were excluded because the electronic medical record was not found (n = 1) and because the time since the last visit to the BHU was superior to three years (n = 9). After compliance with the inclusion and exclusion criteria, the sample was estimate for 108 elderly people.

The data collection involved a household survey conducted by two of the authors and six previously trained scholarship students. A semi-structured was used, as well as a pre-tested questionnaire containing sociodemographic data (sex, age, schooling, income), behavioral data (smoking), clinical data (self-referenced morbidities, MMSE), satisfaction levels (satisfaction with health and satisfaction with access to health services) and quality of care in the PHC (Patient Assessment of Chronic Illness Care - PACIC scores), in its version translated and adapted into the Portuguese language^16^ . Anthropometric data were obtained at participants’ homes and, subsequently, the body mass index was calculated. Blood pressure levels were also measured and biological material was collected to measure the glycemic, lipid profile and renal function parameters. Further details are described in a previous publication^14^ .

After home survey, secondary data from electronic medical records were collected in January 2017. A standard form was filled with information on morbidities; care parameters, including number of medical and nursing consultations and tests (electrocardiogram, fundoscopy, chest X-ray, and diabetic foot evaluation); date of request of the laboratory tests of interest, as well as those examinations requested, but that for some reason were not performed.

The delimitation of the period for analysis of the information on medical records considered the periodicity recommended by the DM and hypertension protocol established by the Minas Gerais State Health Department^17^ .

The procedures of interest and laboratory tests were considered performed if in the tab “Procedure” of the electronic medical record the examination was registered within the recommended interval of time, considering the risk stratification of the participant.

The overall cardiovascular risk classification was categorized as low, moderate, and high risk, according to the protocol of the Municipal Health Department of Belo Horizonte, Minas Gerais^18^ , for its simplicity and ease of adaptation to the municipal resources.

In particular, the degree of compliance to laboratory test requests was calculated by dividing the sum of the laboratory tests in accordance with the above mentioned protocol^17^ by the total number of recommended tests, and expressing it as a percentage. For people with hypertension, the total number of tests recommended was nine and for people with DM, ten tests. Subsequently, they were categorized into: 0% (T1) - (no tests completed); 1-50% (T2) (1 to 4 tests met for hypertension and 1 to 5 tests met for DM); 51-100% (T3) (5 to 9 tests met for hypertension and 6 to 10 tests met for DM).

The quality of care was conceptually supported in two Donabedian dimensions^19^: *process -* consisted in registering the care parameters by the doctor and the nurse; in the degree of compliance to requests for laboratory tests according to risk stratum; and in the number of laboratory tests requested and not performed; and *outcome -* encompassed clinical indicators (control of blood pressure and metabolic levels); perception of the user about the quality of care received in the PHC (PACIC score); degree of satisfaction with health and access to health services; therapeutic goal achieved (results of laboratory tests collected at home).

The biochemical parameters were classified within the range of normality and considered for the analysis of the therapeutic goal achieved: serum creatinine (< 1.3 mg/dL in men and < 1.2 mg/dL in women), microalbuminuria (albumin/creatinine ratio - ACR < 30 mg/g), fasting blood glucose (< 100 mg/dL), total cholesterol (< 200 mg/dL), high-density lipoprotein cholesterol (HDL-c ≥ 40 mg/dL in men and ≥ 50 mg/dL in women), low density lipoprotein cholesterol (LDL-c < 160 mg/dL), triglycerides (< 150 mg/dL), potassium (≤ 5.1 mEq/L), hematocrit (40-50% in men and 36-46% in women), urine routine (absence of abnormal elements and urinary infection) and glycated hemoglobin (HbA1c < 7% in the elderly with DM and < 6.5% in the elderly with hypertension).

Controlled blood pressure (BP-c) was set as blood pressure levels < 140/90 mmHg in the elderly with hypertension and < 130/80 mmHg in the elderly with DM; for high blood pressure (BP-h), values greater than or equal to those mentioned above were considered. Controlled metabolic control (MC-c) was considered HbA1c < 7% in diabetic elderly and < 6.5% in hypertensive elderly, while elderly patients with values outside the above mentioned reference values were considered as altered metabolic control (MC-a).

The degree of satisfaction with health and access to health services was measured by questions number two and 24 of the questionnaire World Health Organization Quality of Life-bref (WHOQOL-bref), translated and validated for the Portuguese language^20^ , respectively. Those individuals who responded being “(very) satisfied” (satisfied with health [SH-s] and satisfied with access [SA-s]) were considered satisfied, and those who were “(very) dissatisfied” or “fairly staisfied”(dissatisfied with health [SH-d] and dissatisfied with the access [SA-d]) were considered dissatisfied.

The PACIC scores were categorized according to the 25, 50 and 75 percentiles, respectively: low (P-l ≤ 1,30), medium (P-m = 1,31-2,22) and high (P-h ≥ 2,23).

Initially, data were analyzed using descriptive techniques expressed as proportions or percentages for categorical variables and median (interquartile range - IQ) for non-parametric continuous variables.

The Multiple Correspondence Analysis (MCA) was used to identify patterns of association between the degree of compliance to the requests for laboratory tests and the parameters of results of the quality of care in PHC. This technique of analysis consists of the graphical projection of the active variables that configure the profiles of the clusters and the supplementary variable that identifies the arrangement of the clusters in a multidimensional design (typology). While looking for the structuring axes, it is essential to observe the effects of the interdependence between the categories of the variables when crossing the axes. From the relational analysis, one can verify in the same space patterns of associations, translated by the definition of several nuclei of homogeneity. The graphical interpretation of the points makes it possible to say that the proximity between the points reflects similarity or association, whereas the distancing of the points is considered non-similar^21^ .

The implementation of the MCA was based on the presence-absence matrix structure of the data, in which the *n* individuals (matrix rows), characterized by *m* attributes, that is, the variables of interest (matrix column) are arranged. Each axis in the graph explains a percentage of the total data variability (inertia)^21^ . For the interpretative process of the MCA, the steps described by Carvalho^21^ were followed. The number of retained dimensions was determined by the magnitude of the eigenvalues and inertia, retaining the dimensions with higher values, that is, the point from which the variance ceases to present marked declines, which, in the study, corresponded to the first two dimensions . The selection of variables for each chosen dimension was guided by the value of the discrimination measure equal to or greater than the value of the inertia. The criterion for selecting the categories of variables for each structuring axis was based on the quantification values (coordinates and contributions). As the sum of the contributions for each dimension is 1, the average contribution was taken as the reference value, which was 0.0714 (1/14 categories). The internal reliability of the retained dimensions was calculated by the Cronbach’s alpha coefficient.

In addition, clusters analysis by the K-means Cluster method was required in order to delimit groupings of individuals with similar characteristics. This technique used the coordinates of the scores (OBject SCOres - OBSCO) determined by the MCA in the most representative dimensions, identifying mutually exclusive clusters by calculating the quadratic Euclidean distance (coefficient of similarity) of the point categories. The validation of the number of clusters obtained was proved by the hierarchical cluster analysis through two distinct clustering criteria (Ward method and criterion of the nearest neighbor). The graphical representation of the fusion coefficients of both methods indicated a marked fall in the distances between the coefficients up to the third cluster, being considered as an optimal cut-off point^21^ .

Complied care parameters (medical and nursing consultations, diabetic foot evaluation, electrocardiogram, funduscopy and chest X-ray examinations) were excluded from the MCA due to the probable underreporting in the medical records, which could compromise the results. The cardiovascular risk variable also did not enter the MCA, considering the existence of multicollinearity between the variables that compose the cardiovascular risk stratification (control of blood pressure and metabolic levels). The removal of this variable from the MCA model resulted in the increase of the inertia values and the quantifications of the two chosen dimensions, obtaining a more consistent pattern of relation between the other variables.

The Statistical Package for the Social Sciences (SPSS, version 23.0, Chicago, IL, USA) was used fot the analysis.

The project was approved by the Research Ethics Committees of the involved institutions (Opinion No. 1,238,099 and Opinion No. 1,351,378), taking into account the legal procedures. The consent form was obtained from each participant and data confidentiality and anonymity were guaranteed.

## Results

The study sample consisted of 108 elderly people, with a median age of 71.5 years (IQ 66.0-81.0 years), predominantly female (n = 75, 69.4%), with up to four years of study (n = 62, 57.4%), and 31.5% (n = 34) had only one minimum wage or less to support the household. More than half of the participants (n = 58, 53.7%) reported having five or more morbidities, of which the most prevalent was dyslipidemia (n = 94, 87.0%) and osteoarthrosis (n = 47, 43.5%). All participants had hypertension (n = 108) and 42.6% (n = 46) had associated DM (data not shown).

The highest percentage obtained in relation to compliance with the recommendations established in the protocol related to the care parameters was the medical consultation item, with a percentage higher than 50% (n = 56). All other items had percentages lower than 30%, of which the worst parameter was the diabetic foot item (n = 1, 2.2%). High cardiovascular risk was present in more than ¾ of the sample (n = 84). Pressure and metabolic levels were controlled in 56.5% (n = 61) and 76.9% (n = 83) of the cases, respectively. More than half of the participants were (very) satisfied with health (n = 62, 59.0%) and with access to health services (n = 65, 61.9%). However, there was worse perception of quality of care ( Table 1 ) with a median PACIC score of 1.55 (IQ 1.30-2.23) (data not shown).

**Table 1:** Distribution of care parameters and results of care for the elderly with hypertension and/or diabetes *mellitus* followed in primary health care. Belo Horizonte, Minas Gerais, Brazil, 2014-2017

Variables	n	%
Care parameters complied		
Medical consultation	56	51.9
Nursing consultation	27	25.0
Electrocardiogram	31	28.7
Fundoscopy	10	9.3
Chest x-ray	10	9.3
Diabetic foot evaluation*	1	2.2
Cardiovascular risk		
Low	14	13.0
Medium	10	9.2
High	84	77.8
Control of blood pressure^†^		
Controlled	61	56.5
Uncontrolled	47	43.5
Metabolic control^‡^		
Controlled	83	76.9
Uncontrolled	25	23.1
Satisfaction with health^§^		
Very satisfied/satisfied	62	59.0
Neither satisfied nor dissatisfied	28	26.7
Very dissatisfied/dissatisfied	15	14.3
Satisfaction with access to health services^§^		
Very satisfied/satisfied	65	61.9
Neither satisfied nor dissatisfied	17	16.2
Very dissatisfied/dissatisfied	23	21.9
% tests complied		
0%	35	32.4
1-50%	24	22.2
51-100%	49	45.4
PACIC scores^||¶^		
Low (≤ 1.30)	29	28.7
Medium (1.31-2.22)	47	46.5
High (≥ 2.23)	25	24.8

*n = 46 (only people with diabetes *mellitus* );^†^Controlled blood pressure = < 140/90 mmHg (elderly patients with hypertension) and < 130/80 mmHg (elderly people with diabetes *mellitus* );^‡^Controlled metabolic control = glycated hemoglobin < 7% (elderly with diabetes *mellitus* ) and < 6.5% (elderly with hypertension);^§^Number of missing information (n = 3);^||^Number of missing information (n = 7);^¶^PACIC = Patient Assessment of Chronic Illness Care

**Figure 1:**
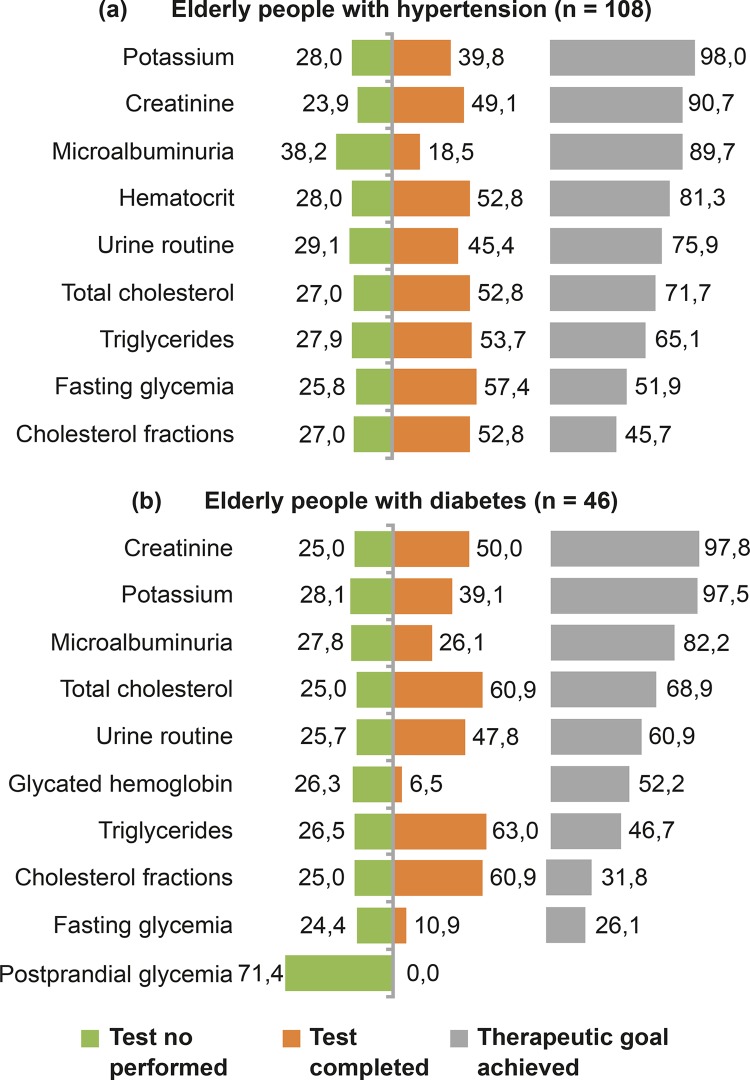
Distribution of laboratory tests in accordance with the parameters recommended by the hypertension (a) and diabetes *mellitus* (b) protocol, unperformed tests and therapeutic goal achieved. Belo Horizonte, Minas Gerais, Brazil, 2014-2017

Analyzing the accomplishment of the actions foreseen in the laboratory protocol, 12 individuals (11.1%) were in agreement with all the recommendations (data not shown), while 35 (32.4%) did not comply with any of the requirements ( Table 1 ). Serum creatinine, routine urine, potassium and microalbuminuria were the analytes with the lowest percentage of compliance in both cases (hypertension and DM) (below 50%); HbA1c, fasting serum glucose and postprandial glycemia for those with DM had null percentages to 10.9%. Among the tests requested and not performed by the participants, microalbuminuria for people with hypertension and postprandial glycemia for those with DM stood out, with percentages of 38.2% (n = 41) and 71.4% (n = 33), respectively. Also, the worst indicator with target therapy achieved below 50% in both cases was found for the analyte cholesterol fractions, whereas serum creatinine and potassium had the best therapeutic goals achieved ( Figure 1 ).

In relation to MCA, after graphical evaluation of the variance of the maximum number of dimensions (r_máx_= 11), the researchers decided to retain the first two dimensions, which were more representative in terms of inertia. Dimension 1 (D1) explained 19.94% ([0.275/1.379] × 100) and dimension 2 (D2), 17.26% ([0.238/1.379] × 100) of the total variance. The internal reliability of D1 and D2 was 0.473 and 0.360, respectively. The bold values shown in Table 2 correspond to the variables that best discriminated in each of the two dimensions under study. All values registered discrimination measures higher than the inertia of the reference dimension.

**Table 2:** Discrimination measures for retained dimensions and percentage of contribution for each variable under study. Belo Horizonte, Minas Gerais, Brazil, 2014-2017

Variables	Dimension 1		Dimension 2	

	Discrimination measures	% contribution	Discrimination measures	% contribution
Metabolic control*	**0.623**	37.8	0.019	1.3
Blood pressure control^†^	**0.429**	26.0	0.129	9.0
Satisfaction with access to health	0.222	13.5	0.115	8.0
Satisfaction with health	0.187	11.3	0.210	14.7
Total PACIC^‡^	0.144	8.6	**0.605**	42.4
% tests complied	0.046	2.8	**0.351**	24.6
No. of cluster^§^	0.711		0.627	
Total active	1.650		1.428	
Inertia	0.275		0.238	
% explained variance	19.94		17.26	
Alpha Cronbach’s	0.473		0.360	

*Controlled glycated hemoglobin = < 7% (elderly with diabetes *mellitus* ) and < 6.5% (elderly with hypertension);^†^Controlled blood pressure = < 140/90 mmHg (elderly with hypertension) and < 130/80 mmHg (elderly with diabetes *mellitus* );^‡^PACIC = Patient Assessment of Chronic Illness Care;^§^Supplementary variable

In this respect, although the variables *satisfaction with health* and *satisfaction with access to health* discriminate less than the other variables in their respective dimensions, they were maintained in the analysis. This decision is supported by three arguments: first, discrimination measures and quantifications (coordinates and contributions) of their categories are not practically null or very low values in both dimensions; secondly, the graphical interpretation of the discrimination measures (data not shown) indicates simultaneity in the composition of the two dimensions (double belonging) because they are located near the diagonal and away from the origin; and third, a more qualitative interpretation, in which the inclusion of these variables contributed to the thematic coherence in the interpretation of the dimensions.

**Figure 2:**
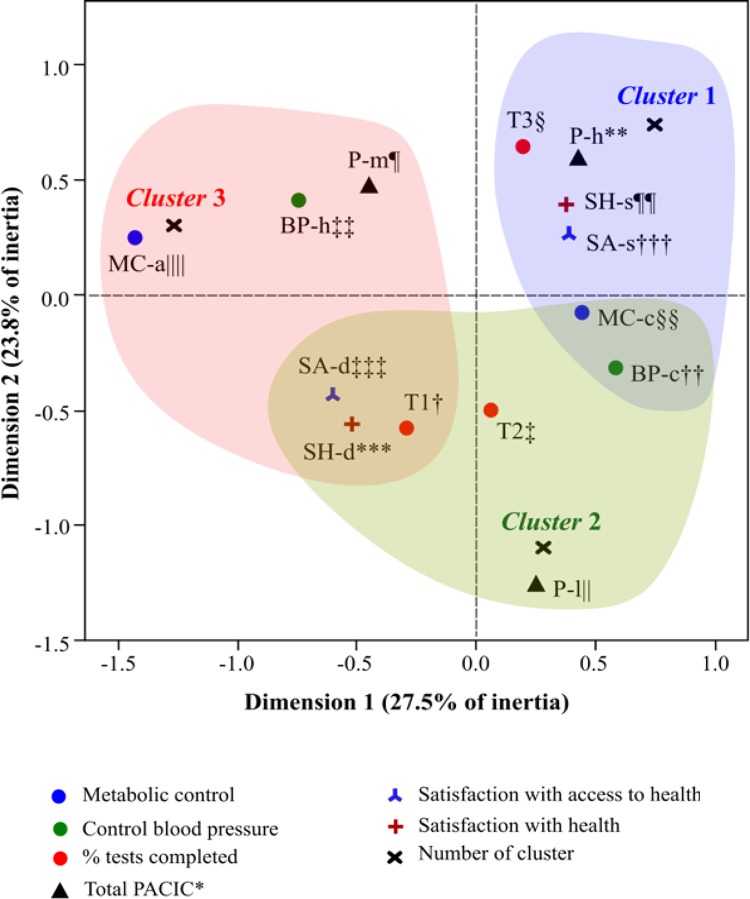
Map of analysis of multiple correspondence according to the degree of conformity to the requests of laboratory tests and parameters of results of the quality of care for elderly people with hypertension and/or diabetes *mellitus* followed in the primary health care. Belo Horizonte, Minas Gerais, Brazil, 2014-2017

Subsequently, the analysis of the quantifications (coordinates and contributions) of the categories of the selected variables identified that D1 was associated with satisfactory control of blood pressure (BP-c) and metabolic levels (MC-c), and with satisfaction with health (SH-s) and with access to health (SA-s) (D1 > 0). In contrast, there are the categories with unsatisfactory control of blood pressure (BP-h) and metabolic levels (MC-a), as well as categories of dissatisfaction with their health (SH-d) and with health access (SA-d) (D1 < 0). In this sense, D1 separates individuals regarding clinical aspects and levels of satisfaction. On the other hand, D2 differentiated individuals with higher percentage of completed tests (T3) and PACIC scores (P-h, P-m), as well as satisfaction with health (SH-s) and with health access (SA-s) (D2 > 0); as opposed to individuals with worse outcome indicators of care (T1, T2, P-l, SH-d, SA-d) (D2 < 0) ( Figure 2 ).

*PACIC = Patient Assessment of Chronic Illness Care.^†^T1 = Completed tests (0%);^‡^T2 = Completed tests (1-50%);^§^T3 = Completed tests (51-100%);^||^P-l = PACIC* low (≤ 1.30);^¶^P-m = PACIC* medium (1.31-2.22); **P-h = PACIC* high (≥ 2.23);^††^BP-c = Controlled blood pressure;^‡‡^BP-h = High blood pressure;^§§^MC-c = Controlled metabolic control;^||||^MC-a = Altered metabolic control;^¶¶^SH-s = Satisfied with health; ***SH-d = Dissatisfied with health;^†††^SA-s = Satisfied with access to health;^‡‡‡^SA-d = Dissatisfied with access to health

In addition, cluster analysis identified the formation of three clusters of individuals with distinct profiles ( Figure 2 ). Cluster 1 obtained the highest number of individuals, with 37.0% (n = 40) of the sample, followed by cluster 2 (n = 36, 33.3%), and cluster 3 (n = 32, 29.6%). The first cluster consisted of individuals with better indicators of quality of care, evidenced by compliance with laboratory tests above 50% (T3), elevated PACIC score (P-h), control of blood pressure (BP-c) and metabolic levels (MC-c), and satisfaction with health (SH-s) and with access to health (SA-s). In contrast, cluster 3 was constituted by a complete absence of compliance with guidelines (T1), median PACIC score (P-m), elevation of blood pressure (BP-h) and metabolic levels (MC-a), and dissatisfaction with health (SH-d) and with access to health (SA-d). The other profile presented in this study (cluster 2) was characterized by individuals with stable clinical indicators (BP-c and MC-c), but with compliance rates below 50% (T1 and T2), low PACIC score (P-l) and who are dissatisfied with health (SH-d) and with access to health (SA-d) ( Table 3 ).

**Table 3:** Characterization of clusters according to results of care for the elderly with hypertension and/or diabetes followed in primary health care. Belo Horizonte, Minas Gerais, Brazil, 2014-2017

Variables		Typology					
	
		Cluster 1		Cluster 2		Cluster 3	
	
		n	%	n	%	n	%
% tests completed	T1^†^(0%)	7	17.5	15	41.7	13	40.6
	T2^‡^(1-50%)	3	7.5	14	38.9	7	21.9
	T3^§^(51-100%)	30	75.0	7	19.4	12	37.5
	Total	40	100.0	36	100.0	32	100.0
Total PACIC*	P-l^||^	3	8.3	23	69.7	3	9.4
	P-m^¶^	16	44.4	9	27.3	22	68.8
	P-h**	17	47.2	1	3.0	7	21.9
	Total	36	100.0	33	100.0	32	100.0
Control of blood pressure	BP-c^††^	28	70.0	30	83.3	3	9.4
	BP-h^‡‡^	12	30.0	6	16.7	29	90.6
	Total	40	100.0	36	100.0	32	100.0
Metabolic control	MC-c^§§^	38	95.0	35	97.2	10	31.3
	MC-a^||||^	2	5.0	1	2.8	22	68.8
	Total	40	100.0	36	100.0	32	100.0
Satisfaction with health	SH-s^¶¶^	37	92.5	14	41.2	11	35.5
	SH-d***	3	7.5	20	58.8	20	64.5
	Total	40	100.0	34	100.0	31	100.0
Satisfaction with access to health	SA-s^†††^	36	90.0	15	44.1	14	45.2
	SA-d^‡‡‡^	4	10.0	19	55.9	17	54.8
	Total	40	100.0	34	100.0	31	100.0

*PACIC = Patient Assessment of Chronic Illness Care;^†^T1 = Completed tests (0%);^‡^T2 = Completed tests (1-50%);^§^T3 = Completed tests (51-100%);^||^P-l = PACIC* low (≤ 1.30);^¶^P-m = PACIC* medium (1.31-2.22); **P-h = PACIC* high (≥ 2.23);^††^BP-c = Controlled blood pressure;^‡‡^BP-h = High blood pressure;^§§^MC-c = Controlled metabolic control;^||||^MC-a = Altered metabolic control;^¶¶^SH-s = Satisfied with health; ***SH-d = Dissatisfied with health;^†††^SA-s = Satisfied with access to health;^‡‡‡^SA-d = Dissatisfied with access to health

## Discussion

This study, including the elderly with DM and/or hypertension followed in PHC, showed low conformity of the care practice according to stratification of cardiovascular risk and metabolic control, which affects the quality of care achieved. Also, three clusters of individuals with distinct profiles were identified, being the first and third cluster formed by the elderly that concentrated the best and the worst quality indicators of the care, respectively.

Even with different methods, which limit the comparison between the data obtained, other national and international studies also emphasized the incipience of the use of the clinical protocol of DM and hypertension recommended for the improvement of the assistance by physicians and nurses working in PHC^10 - 11 , 22 - 23^ .

In this sense, challenges still need to be overcome to ensure improved care for people with chronic health conditions. Mainly regarding the periodicity of laboratory tests, the present study identified percentages that were far below those recommended by the protocol, especially those related to the glycemic profile in the elderly with DM. This finding is against one of the specific objectives of the Health Care Network for People with Chronic Diseases, which includes providing access to adequate diagnostic and therapeutic resources in a timely manner, guaranteeing the comprehensiveness of care, according to the health needs of the user^24^ .

In general, there was more compliance to hypertension recommendations when compared to DM recommendations, whose highest proportion was found for fasting blood glucose (five times more). This data indicates that the comprehensiveness of care may not yet constitute a priority in the care of the users in the UBS investigated, which may justify the low percentage of people with adequate metabolic control, as verified in the study. In addition, postprandial glycemia was not fulfilled in any of the cases investigated, and therefore, it is not a routine examination of the glycemic profile evaluation and follow-up of prescribed drug therapy.

Part of this non-compliance can be attributed to the absenteeism of the elderly themselves to the laboratory tests, which deserves to be discussed. This situation not only contributes to the progressive growth of repressed demand, reducing the possibility of access, but also allows for an increase in the costs of care, since the postponement or non-performance of the requested tests may result in the complication of user’s condition^25^ .

In the literature, there have been successful experiences on monitoring the absenteeism rate, such as those using information systems to identify the user in detail and the organization of the services. They also include monitoring of individual care targets and rate of service utilization. However, for such a strategy to be translated into practical results, it is necessary for the information system to promote the aggregation of the clinical data of the users, in order to assist the planning process, the recognition of risk groups, with special needs, and the management of comprehensive care, including the issuance of warnings and alerts for the maintenance of bond with the user^23^ .

Regarding the nursing consultation, there was a low systematic record about this care practice in most of the medical records, being below that defined by the protocol (minimally performed semiannually)^17^ . As an aggravating circumstance, there was centralization of the records on the complaint-behavior during the reception, with consequent referral to medical evaluation. This, therefore, underestimated the skills and the specific attributions of the nurse in the care of the elderly who experience a chronic health condition, as already found in another research^22^ .

This finding may be the result of different factors, such as the nurses’ academic training process, which prioritizes in the curriculum the nursing consultation of children and pregnant women, the working conditions, the incomplete nursing records, and the professional’s own posture, as evidenced in Brazilian studies^26 - 27^ . This situation may contribute to the nursing category losing their already achieved role within the Family Health teams, with consequent withdrawal of the clinical practice of this professional in the care for the elderly, which will be increasingly in charge of the physician, thus reinforcing the physician-centered model.

In addition, the results also pointed out problems concerning the medical consultation, despite presenting better results when compared to the nursing consultation. A percentage of 34.3% of the total number of medical records investigated did not contain any or only one record of medical consultation (data not shown), whereas the protocol^17^ recommends two to three annual consultations, depending on the cardiovascular risk in people with hypertension or on the metabolic control in people with DM.

In view of the situation verified, there is a potential lack of supply or poor management in the scheduling of these consultations, which goes against the proposals for implementation of the Health Care Network that recommends the approximation of epidemiological surveillance with the scenario of occurrence of chronic conditions, with a view to strengthening risk stratification. Thus, the aim is to guarantee management based on the health needs of the population^28^ , taking into account one of the most important principles of the SUS, namely, equity^13^ .

In this sense, there are likely to be gaps in the organization of the work process of the Family Health teams, so as to offer an equal and comprehensive care, welcoming the users and linking them to the services offered according to their real needs. This is because when a given population is not stratified by risks, there may be an undersupply of necessary care to those at greater risk and/or over-provision of unnecessary care to those with lower risks, resulting in ineffective and inefficient care. This problem explains, in large part, the difficulties of opening the agenda in the Family Health Strategy for health care to people with chronic conditions with care that does not benefit people^13^ .

The present study also revealed a low percentage in the requests for imaging tests. However, the presence of possible failures in the communication between the care points in the network cannot be ruled out, which may result in underreporting of these procedures that are performed in other levels of care. This assertion is lacking in evidence, but if it is proven, managers need to prioritize this issue in order to guarantee counter-referral in the network, considered essential for the coordination of PHC care. For some authors^13 , 22 , 28^ , this communication between the different points of care of the network will give rationality to the service, avoiding that tests are performed in duplicate or that are requested unnecessarily, optimizing resources with impact on the resolubility.

On the other hand, the identification of the three clusters in the present study emphasized that, although it is widely recognized the importance of standardizing the clinical and therapeutic behavior for people with hypertension and DM in order to achieve better clinical and functional results, this can not always be be evidenced. One possible explanation stems from the very complexity of care for the person who experiences a chronic health condition, since it requires management in a proactive, continuous and integrated way by the health care system, professionals and users/family for effective, efficient, and quality control^13^ . Therefore, compliance with the recommendations found in the protocol alone does not necessarily guarantee better results as observed in the different clusters identified in the study.

In particular, clusters 2 and 3 presented low compliance with the protocol recommendations, however the second cluster agglomerated elderly with controlled clinical parameters, unlike the third cluster. This suggests the presence of other variables that may contribute to this finding not contemplated in the present analysis, for example, a higher concentration of older people with better self-care in cluster 2 than in cluster 3. Such assertion lacks evidence and demands further investigation.

The data presented in this research reinforce the importance of raising awareness and qualifying Family Health teams to improve the implementation of guidelines in clinical practice to favor greater control of elderly people with DM and/or hypertension in order to promote the alignment of health care with the health needs of the population at different risk strata. In order to do so, we encourage the creation of partnerships with higher education institutions that can serve as support, exploring the region’s resource of tele-health, which is currently coordinated by a major higher education institution in the capital, enabling a support and continuing education, as highlighted in the guidelines of the National Policy for Permanent Education in Health^24^ .

In addition, it is important to highlight the role of specialized ambulatory care units that are part of the network, whose performance is not limited only to their assistential role, but also in the supervision and permanent education of PHC professionals. From this perspective, it is necessary to identify which professionals have these skills developed and, once identified, provide the necessary means to organize the professionals’ agenda in order to guarantee time and space for these activities. Previous experiences show that specialized professionals are crucial in the permanent education of general practitioners^13^ .

In addition, this study may support the nurse’s role, motivating them to play the role of articulator of the work process in the Family Health Strategy, helping to rethink the provision of care to the elderly who experience a chronic health condition, encouraging the performance of a multidisciplinary team truly committed to an equitable, comprehensive, and resolutive practice. In this context, not only nursing, but all professionals should fully accomplish their duties and competencies, recording them in medical records, valuing the information contained in these documents, thus improving the quality of the information generated.

Finally, this study reinforces the need to institutionalize health assessment policies as a process of transforming PHC practices and strengthening those already existing in Brazil, such as Administrative Rule no. 483, of April 1, 2014^24^ , that in redefining the Health Care Network of People with Chronic Diseases in the SUS and establishing guidelines for the organization of their lines of care, recommends the monitoring and evaluation of the quality of services through structure, process and performance indicators that investigate the effectiveness and resolubility of care.

Periodic clinical audits, as well as feedback with health professionals involved in care from the dissemination of reports with the data and indicators monitored is a strategy that can effectively systematize these policies^29^ .

The results of the study should be interpreted with caution because of its transversal design, which makes it impossible to establish a temporal and causal relationship between care parameters and the quality of care in PHC. Another limitation refers to the secondary data obtained from electronic medical records, which depends on the quality of health professionals’ records (information bias) and the type of analysis applied, without any control of the confounding factor. However, these limitations do not make the findings unviable, rather, they may help managers and health professionals in strengthening policies aimed at assessing the quality of care in PHC.

## Conclusion

The study showed low conformity of care practice, with emphasis on the evaluation of diabetic foot and the request of specialized tests, indicating failures in the process of care in PHC. It was also verified that the clustering technique proved to be interesting as a clinical management tool, allowing the identification of distinct groups within the same health service, consequently directing specific interventions.

It was also possible to confirm the existence of asymmetries between health care provision by the Family Health teams and the needs presented by the elderly with DM and/or hypertension, presenting a less favorable scenario for the segment with worse care results.
